# Annotations

**Published:** 1896-09-26

**Authors:** 


### ANNOTATIONS.

### Death Rates and Salubrity.

IT is an extremely interesting fact that there are districts within the confines of London in which, even after all correction has been made for the immigration of young adults and the proportion of the sexes, the death-rate is far lower than it is as an average over England and Wales. If we take to the two extremes we find that, while St. George-in-the-East has a corrected death-rate of 27·4, the Strand of 26·8, Limehouse of 26·9, and St. George, Southwark, 25·5, Wandsworth has one of only 13·2, Hampstead 12·2, and Stoke Newington the surprisingly low rate of 10·5. There are many causes which co-operate in producing these great differences, among which may be mentioned the varied incidence of poverty, over-crowding, drink, and injurious trades, all of which tend to increase the death-rate in the districts where they are more than usually present. But when we come to the other side of the question, and try to conceive what health conditions can result in a death-rate of only 10·5 per thousand, after all allowance is made for age and sex, we are bound to confess that such a death-rate, which on the hypothesis that it is due to health conditions would mean a longevity quite unprecedented in the history of recent times, involves the presence of factors quite outside the range of the observations of our statisticians, so far as they have carried their researches at present. This, of course, carries with it come serious consequences. If the death-rate of 10·5 per thousand cannot be explained by health conditions, a proposition which we think cannot be seriously disputed, how about the opposite? How about the assertions so constantly made that the corrected rate of 27·4, for example, which obtains in St. George-in-the-East, is due to unhealthy conditions which are in large part removable? Beside sanitary surroundings there is a social sorting out of the population which is probably almost as potent a factor as are health conditions in the making of a death-rate, and until these are worked out far more completely than they are at present, it is premature to speak so strongly as some do in regard to the healthiness or unhealthiness of any place on the basis even of its corrected death-rate.

### The Influence of Mountain Air.

IT is a matter of no small interest to that large number of *poitrinaires* who annually betake themselves to the mountains for the winter months, to ascertain how the benefit is brought about which they undoubtedly receive. By many the mechanism of the Alpine cure has been is thought to be a mere matter of physics. Recognising that phthisis is chiefly prone to occur among those who from their mode of life do not thoroughly expand their lungs, and that in the rarer atmosphere of high altitudes a much more complete expansion of the lungs is necessary, to obtain the same quantity of oxygen, than would be required in the denser air of the plains, the benefit of residence in the mountains has been attributed to a constant unconscious effort to expand the lung more fully, and to the consequent greater interchange of air in its recesses. Certainly the marked enlargement of the capacity of the chest which is often found after a few months' residence in the high Alps, and the tendency to the development of compensatory emphysema around the diseased and contracting portions of the lungs, tend to show that this theory is correct so far as it goes. But all who go into the Alps feel that there is something more than this in mountain air, and it has recently been shown that not only do the chest muscles adapt themselves to the new conditions, but that the blood itself becomes altered in response to the lessened amount of the more rarified oxygen which each blood corpuscle can carry. The effect of this process of adaptation is that the red corpuscles multiply considerably while, according to some, the hæmoglobin is also increased. In any case, the oxygen-carrying capacity of the blood is increased. This accords fully with the well-known effect of mountain air in the treatment of anæmia, and also with the feeling of well-being felt by visitors to high altitudes. It does not do, however, to dissect a climate too much, or to attribute to one or two factors what really is due to the concurrence of many influences, and it seems probable that mountain air depends for its efficacy on many conditions besides its lessened density, such, for example, as its purity, its dryness, and its comparatively low temperature, combined with the intense solar radiation common at high altitudes. That these are the really important conditions, and that lowered barometric pressure is not everything, is shown by the fact that to get the full benefit of the mountains people must get out of doors, whereas the lessened density of the air should affect those indoors just as much as those outside. Nevertheless we are quite prepared to believe that the compensatory blood changes produced by residence in the mountains are important elements in the "mountain cure."

### The Surgical Uses of Worsted.

AS anyone knows who has carefully observed the difference between a cotton and a woollen shirt, the fibre of the wool remains elastic, and the fabric remains open even when it is wet, whereas cotton fibre under the influence of moisture becomes flabby, and the air is driven out from the interstices of the fabric, which becomes sodden and limp. In surgery gauze is much used as a means of draining cavities, and cotton wool is also much used in place of sponges. The inherent tendency, however, of cotton fibre to lose its elasticity when wet very much interferes with the usefulness of all derivatives of cotton for the purposes mentioned. The iodoform gauze used for drainage has to be employed in considerable mass, the drainage really taking place between the layers rather than in the substance of the fabric, and as for the cotton wool sponges, unless they are puffed out by some more elastic fibre, such as tow or other coarse material, they become, when wet, a flabby, soapy-feeling mass, with very little "wiping" power. Dr. Harold Stiles, of Edinburgh, advocates the use of worsted for both these purposes. The material used is what is known in the trade as white double Berlin wool. For purposes of drainage it is boiled, wrung out of one in a thousand solution of corrosive sublimate, cut into lengths of about eighteen inches, and, with disinfected hands, rubbed in sterilised iodoform. For making sponges it is cut into short pieces and tied up in thin gauze. These, he says, are almost as elastic as ordinary sponges, and are infinitely better than pieces of gauze. We may add that at some hospitals flat pads of closely-knitted worsted are used in laparotomy instead of the flat sponges which are so commonly employed.

